# *Castanea sativa* Mill. Shells Aqueous Extract Exhibits Anticancer Properties Inducing Cytotoxic and Pro-Apoptotic Effects

**DOI:** 10.3390/molecules24183401

**Published:** 2019-09-19

**Authors:** Nunzio Antonio Cacciola, Giuseppe Squillaci, Mariella D’Apolito, Orsolina Petillo, Francesco Veraldi, Francesco La Cara, Gianfranco Peluso, Sabrina Margarucci, Alessandra Morana

**Affiliations:** 1 Research Institute on Terrestrial Ecosystems (IRET), National Research Council of Italy, (CNR), Via P. Castellino 111, 80131 Naples, Italy; nunzioantonio.cacciola@unina.it (N.A.C.); giuseppe.squillaci@iret.cnr.it (G.S.); maria.dapolito@cnr.it (M.D.); orsolina.petillo@cnr.it (O.P.); fr.veraldi@gmail.com (F.V.); francesco.lacara@cnr.it (F.L.C.); gianfranco.peluso@cnr.it (G.P.); 2 Department of Veterinary Medicine and Animal Productions, University of Naples - Federico II, Via F. Delpino 1, 80137 Naples, Italy; 3 Department of Precision Medicine, University of Campania “Luigi Vanvitelli”, Via Santa Maria di Costantinopoli 16, 80138 Naples, Italy

**Keywords:** chestnut shells, polyphenols, bioactive compounds, apoptosis, cytotoxicity, human cell lines

## Abstract

In this study, chestnut shells (CS) were used in order to obtain bioactive compounds through different extraction procedures. The aqueous extracts were chemically characterized. The highest extraction yield and total phenolic content was obtained by conventional liquid extraction (CLE). Gallic and protocatechuic acids were the main simple phenols in the extract, with 86.97 and 11.20 mg/g chestnut shells dry extract (CSDE), respectively. Six tumor cell lines (DU 145, PC-3, LNCaP, MDA-MB-231, MCF-7, and HepG2) and one normal prostate epithelial cell line (PNT2) were exposed to increasing concentration of CSDE (1–100 µg/mL) for 24 h, and cell viability was evaluated using 3-(4,5-dimethylthiazole-2-yl)-2,5-diphenyltetrazolium bromide MTT assay. A reduced rate in cell viability was observed in DU 145, PC-3, LNCaP, and MCF-7 cells, while viability of the other assessed cells was not affected, except for PNT2 cells at a concentration of 100 μg/mL. Furthermore, CSDE—at concentrations of 55.5 and 100 µg/mL—lead to a significant increase of apoptotic cells in DU 145 cells of 28.2% and 61%, respectively. In conclusion, these outcomes suggested that CS might be used for the extraction of several polyphenols that may represent good candidates for alternative therapies or in combination with current chemotherapeutics.

## 1. Introduction

The plant kingdom produces a wide number of phytochemicals, known as secondary metabolites. Interestingly, polyphenols are the most abundant class of compounds belonging to this group. They are largely present in the plant tissues and are synthesized as defense agents against damaging events, such as predators or ultraviolet light [1]. In addition, the well-established antioxidant power of the phenolic compounds is responsible for a variety of biological effects that contribute to benefits on human health [2]. During the cell metabolism, potentially harmful products, known as reactive oxygen species (ROS), are produced in the human body. They react at a high rate with almost every type of organic molecule present in the living cells, thus creating damages and leading to disease conditions [3]. Phenolic compounds, acting as ROS scavenger and chain breakers of the radical polymerization reaction, concur to the prevention of human diseases associated with oxidative stress [4]. They may reduce blood pressure and inhibit platelet aggregation [5,6], decrease the oxidation of LDL with positive action on cardiovascular diseases [7], and protect against neurodegenerative disorders [8]. Phenolic compounds can also exert anticancer activity: in fact, in vitro and in vivo studies demonstrated the pivotal role of curcumin, resveratrol, quercetin, and epigallocatechin gallate contained in turmeric, red grape, and green tea, respectively, in several steps of the carcinogenesis process [9,10]. An anti-proliferative effect on MDA-MB-231 human breast cancer cells was shown by flaxseed oil, mainly composed of vanillic, ferulic, and *p*-hydroxybenzoic acids. The same extract exhibited both cytotoxic and pro-oxidant activity against MCF-7 cells [11]. Nowadays, the research on phytochemicals is actively increasing, with the aim of identifying new molecules or blends of them to use in cancer prevention and/or treatment [12]. In this perspective, the exploration of novel sources of natural compounds has been encouraged, and the large amount of agro-industrial wastes generated by the anthropic activities could represent an exploitable and valuable resource for the production of bioactive molecules [13,14]. *Castanea sativa* Mill. (sweet chestnut) is recognized as one of the most remarkable trees in the world due to its economic importance. A large part of chestnut fruit is used for fresh consumption and for preparation of products such as purée, marron-glacé, and flour. The chestnut processing chain generates large amount of wastes (burs, shells, and leaves)—generally discarded—but still rich in bioactive compounds. In fact, chestnut leaves are used as an infusion in folk medicine for curing diarrhea and coughs [15], and several reports describe the antioxidant potential of phenolic compounds from burs and shells [16,17]. In 2017, Italy, the main chestnut producer in the European union, providing 38% of the total European chestnut production, yielded about 52,356 tons (FAOSTAT, food and agriculture organization of the United States: http://www.fao.org/faostat/en/#data accessed on 17 July 2019). The chestnut peeling process generates a large volume of outer and inner shells representing approximately 10% by weight of the whole chestnut [18]. Currently, factories burn this residue to overcome disposal problems, but it could represent an attractive solution for the production of biologically active compounds from a cheap and underexploited source. In fact, outer and inner chestnut shells (CS) contain 2.7–5.2% (*w*/*w*) of phenolic compounds, which exhibit antioxidant activity [19], and about 36% (*w*/*w*) of sugars that can be used as feedstock for biofuels production [20]. The role of the phenolic compounds from CS as anticancer agents is largely reported: a blend of monomers, dimers, and trimers of procyanidins from *Castanea mollissima* Bl CS extract, and a hydroalcoholic shell extract from *Castanea sativa* Mill. exhibited cytotoxic effects on human hepatoblastoma Hep G2 cells [21,22], whereas extracts obtained from chestnut (*Castanea crenata*) inner shells showed anticancer effects towards the following human cancer cell lines: HeLa (cervical cancer cell line), AGS (gastric cancer cell line), LoVo, and HT-29 (colon cancer cell lines), MCF-7 (human estrogen-receptor-positive breast cancer cell line), and Hep G2 cells (liver carcinoma cell line) [23]. In this study, bioactive compounds deriving from CS were extracted through three eco-friendly methods (conventional liquid extraction, CLE; ultrasound assisted-extraction, UAE; microwave-assisted extraction, MAE), and the effectiveness of the extractions were assessed by the estimation of the total phenol, *ortho*-diphenol, flavonoid, and tannin contents in the extracts. In light of the above information, we aimed to evaluate the possible biological effects of the chestnut shell dry extract (CSDE) from CLE on human normal and cancer cell lines.

## 2. Results

### 2.1. Characterization of the Phenolic Compounds from CSDE

In the present work, three aqueous extracts were obtained from burned CS by means of different procedures. CS (5 g) were extracted for 1 h, and 260, 111, and 191 mg of CSDE were obtained by CLE, UAE, and MAE, respectively. The CLE gave the highest extraction yield and total phenolic content, with 5.2 ± 0.1% (mg CSDE/g CS) and 312.44 ± 3.32 mg gallic acid equivalents (GAE)/g CSDE, respectively (Table 1).

The UAE was the less effective method with extraction yield and total phenolic content of 2.2 ± 0.1% and 190.12 ± 1.16 mg GAE/g CSDE, respectively. As *ortho*-diphenols are the class of phenolic molecules endowed with a strong antioxidant power due to the position of the two hydroxyl groups on the aromatic ring, it was decided to determine their content. The presence of the hydroxyl groups in *ortho* position (catechol structure) increases the antioxidant activity of the compounds through the stabilization of the phenoxyl radical [24,25]. The *ortho*-diphenols in the extracts were very variable, with the CLE extract containing an amount 2-folds higher than that of the UAE extract (148.72 ± 2.61 mg caffeic acid equivalents, CAE/g CSDE, and 73.90 ± 1.01 mg CAE/g CSDE, respectively). Tannins were the main class of phenolic compounds contained in the three extracts and varied from 118.97 ± 2.12 (UAE) to 205.99 ± 1.95 (CLE) mg GAE/g CSDE. Also, flavonoids were present in greater quantities in the CLE extract, and their amount followed the trend of the other phenolic families: CLE > MAE > UAE.

The Folin–Ciocalteu method is the most common method used for the estimation of the total phenols in natural extracts, but it does not provide information about the type of phenolic molecules. By means of Reversed Phase-High Performance Liquid Chromatography (RP-HPLC) analysis, some phenolic compounds were identified in the extracts (Table 2).

More in detail, the following molecules: gallic acid (GA), protocatechuic acid (PCA), chlorogenic acid (CGA), epicatechin (EC), ellagic acid (EA), *p*-coumaric acid (*p*CA), syringic acid (SyA), sinapic acid (SA), ferulic acid (FA), and scopoletin (S) were identified in the three extracts by comparison of their retention times with those of the available standards. GA was the most abundant compound identified, and its amount was comprised between 86.97 ± 1.32 mg/g CSDE (CLE) and 150.09 ± 2.16 mg/g CSDE (UAE). PCA was the second most represented phenolic compound, with concentrations ranging from 11.20 ± 0.30 mg/g CSDE (CLE) to 21.57 ± 1.57 mg/g CSDE (UAE). Minor phenolic compounds were also detected, and among them, CGA, EP, and EA were the main representatives, with the highest concentrations in MAE (1.18 ± 0.04 mg/g CSDE, 1.28 ± 0.02 mg/g CSDE, and 1.09 ± 0.03 mg/g CSDE, respectively). The collected data showed that the extraction from CS by boiling water (CLE) provided the highest extraction and phenolic yields. Moreover, the qualitative molecular profile of the compounds identified by RP-HPLC was similar to that of the extracts obtained by UAE and MAE procedures. Thus, according to this, the CLE extract was selected for tests on human cancer cell lines.

### 2.2. CSDE Exerts Cytotoxic Effects on Human Cancer Cell Lines 

Cytotoxic effect of CSDE was assessed in three prostate cancer cell lines (PC-3, DU 145, and LNCaP), two breast cancer cell lines (MCF-7 and MDA-MB-231), Hep G2 (hepatocellular cancer cell), and a non-cancerous prostate cell line (PNT2). Cells were treated with increasing concentrations of CSDE (1-3-10-17.5-30-55.5 and 100 μg/mL) for 24 h and the cytotoxic effect of CSDE on cell viability was assessed by MTT assay. Results showed that CSDE possesses cytotoxic effect on DU 145, PC-3, LNCaP, and MCF-7 cancer cells, while no cytotoxic effect was observed in MDA-MB-231, Hep G2, and PNT2 cells. 

**Figure 1:**
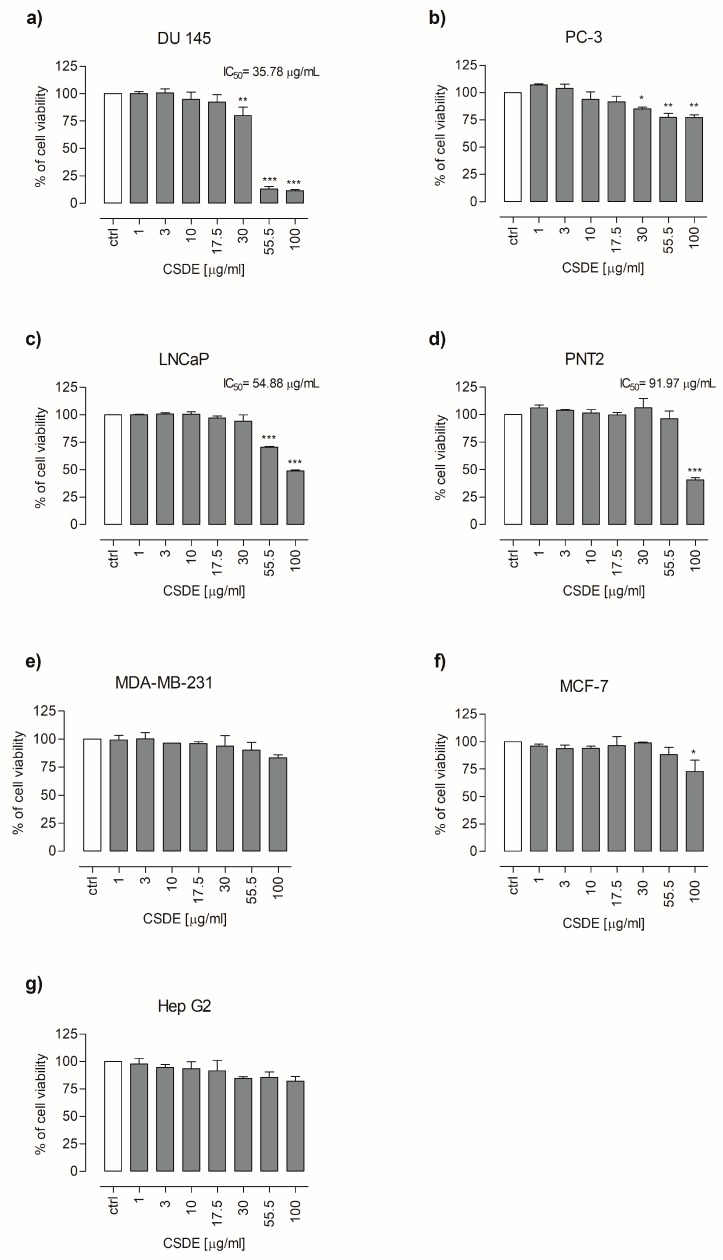
Cytotoxic effects of chestnut shells dry extract (CSDE, 1–100 µg/mL, 24 h exposure) in DU 145 (**a**), PC-3 (**b**), LNCaP (**c**), PNT2 (**d**), MDA-MB-231 (**e**), MCF-7 (**f**), and Hep G2 (**g**) cells. Viability (expressed as percentage of cell viability) rate was investigated by using the MTT assay. Each bar represents the mean ± SD of three independent experiments. * *p* < 0.05, ** *p* < 0.01, and *** *p* < 0.001 vs. control (untreated cells).

In particular, the results showed that CSDE at concentrations of 30, 55, and 100 µg/mL reduced the viability (IC_50_ = 35.78 ± 0.029) of DU 145 cells by 20, 87, and 89%, respectively (Figure 1a). At the same concentrations, CSDE diminished the viability of PC-3 cells by 14.9, 22.8, and 23%, respectively (Figure 1b). Furthermore, CSDE at concentrations of 55.5 and 100 µg/mL also diminished the viability (IC_50_ = 54.88 ± 0.026) of LNCaP cells by 29% and 51%, respectively (Figure 1c). No cytotoxic effect on the viability of PNT2 cells was observed, except at 100 µg/mL where cell viability diminished by 58% (Figure 1d). As far as breast cancer cells are concerned, the extract did not affect the viability of MDA-MB-231 cells (Figure 1e) while CSDE, at the concentration of 100 µg/mL, reduced the viability of MCF-7 cells by 27% (Figure 1f). CSDE, at any concentration tested, did not affect the viability of Hep G2 cells (Figure 1g).

### 2.3. CSDE Induces Apoptosis in DU 145 Cancer Cell Lines

**Figure 2:**
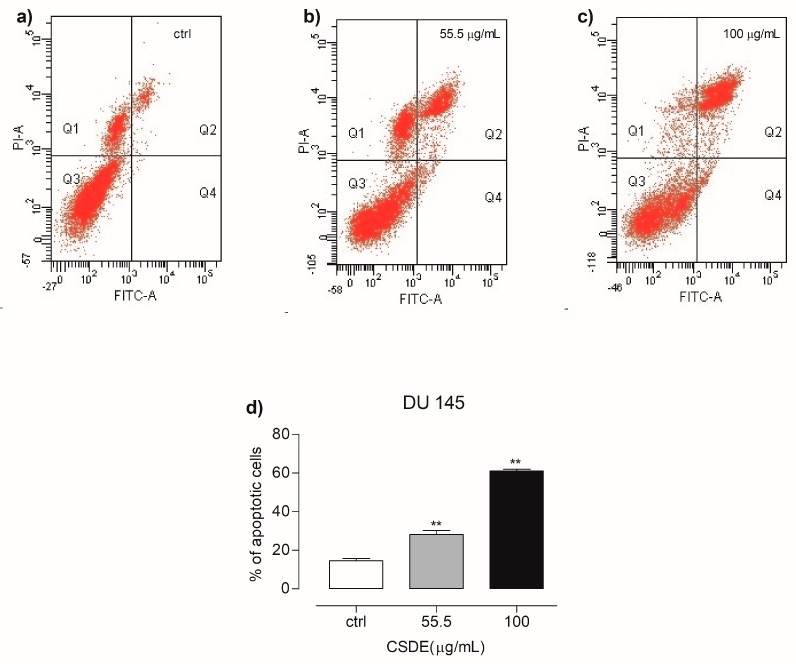
Representative dot plots showing cells in necrosis (Q1), late apoptosis (Q2), live cells (Q3), and early apoptosis (Q4), for control (**a**), 55.5 µg/mL (**b**) and 100 µg/mL (**c**), upon exposure with CSDE for 24 h. (**d**) Graph shows the number of cells undergoing both early and late apoptosis. Details are described in the materials and methods section. Each bar represents the mean ± SD of three independent experiments. ** *p* < 0.01 vs. control (untreated cells).

Since DU 145 cells were found to be the cells with higher sensitivity to CSDE treatment with respect to other cell lines tested, we decided to evaluate whether the reduced cell viability was correlated to apoptosis processes. To this end, we carried out flow cytometry analysis by using Annexin V/PI apoptosis assay. As depicted in Figure 2, the treatment with 55.5 or 100 μg/mL of CSDE for 24 h led to a significant increase in late apoptosis rate, corresponding to 28.2% and 61% respectively, compared to untreated cells at 14.1%. (Figure 2a–d). In Figure 2d, we reported the percentage of cells in late apoptotic phase.

## 3. Discussion

Chestnut shells (CS) used in this work are an industrial by-product derived from the “*Brulage*” peeling process. This process consists of heating chestnuts up to 900 °C for a few seconds with production of a blend of outer and inner shells. Since the yield and quality of bioactive compounds are usually different in relation to the technique and operative parameters used, we compared the polyphenols extraction efficiency by using three different methods (CLE, UAE, and MAE) [26]. All the extractions were carried out avoiding organic solvents as the use of eco-compatible procedures has increased in recent times due to environmental, health, and safety concerns. They were performed using the same solvent in order to ensure that any difference among the tested methods was independent of the solvent used. Water was selected as the extraction solvent for its non-toxic character, low cost, environmental impact, and its safety in all the health care applications.

UAE and MAE were tested because, nowadays, they are techniques frequently applied for the recovery of organic compounds. UAE is one of the most economical extraction methods and has less instrumental requirements [27], while MAE has the following advantages: ease of handle, fast heating of the solvents, and short application times [28].

However, the extraction yields and the phenolic compounds recovery by MAE are sometimes low in comparison to other extraction methodologies. Fernandez-Agullò et al. obtained a lower phenolic recovery from *Castanea sativa* CS by means of this technique compared to the conventional extraction. The total phenolic contents were 51.71 ± 1.54 and 56.23 ± 2.44 g GAE/100 g extract after extraction in water at 50 and 75 °C, respectively, whereas 33.39 ± 1.86 and 33.88 ± 1.62 g GAE/100 g extract were obtained at the same temperatures by MAE [29]. According to this, under our experimental conditions MAE treatment produced an extract with lower phenolic content with respect to the CLE. The highest extraction and phenolic yields reached by CLE were probably due to the agitation of the mixture coupled with the high temperature. In fact, the stirrer speed is a significant factor to take into consideration because the vigorous agitation facilitates the contact between solvent and CS, thus improving the bioactive compounds extraction [30].

The lowest extraction and phenolic yields obtained through UAE were likely due to the low temperature used in the process (60 °C). In fact, it is described that sometimes the lower the temperature, the lower the release of the phenolic compounds [31].

However, it must be underlined that the high temperature can also negatively affect the yield of some molecules, thus changing the composition of an extract. The increase of the phenolic yield by CLE (Table 1) does not necessarily imply a concomitant increase of all molecules contained in the extract (Table 2). Small molecules, such as gallic acid and protocatechuic acid for instance, can be more sensitive to the high temperature with respect to flavonoids or tannins, and some degradation and/or oxidation phenomena may have occurred.

Polyphenols are phytochemicals provided with powerful biological actions, including the anticancer activity [32]. This important effect is mainly ascribed to low molecular weight polyphenols, such as those belonging to the phenolic acid and flavonoid classes [33,34]. For this reason, we decided to assess the possible potential anticancer effect of chestnut shell dry extract (CSDE) on six human cancer cell lines (DU 145, PC-3, LNCaP, MDA-MB-231, MCF-7, and Hep G2) and one normal human prostate cell line (PNT2). The pharmacological treatment with CSDE was able to inhibit cell viability of different cancer cell lines (DU 145, PC-3, LNCaP, and MCF-7) and, therefore, we decide to investigate more in-depth the effect underlying the cytotoxicity in DU 145 cells, since the latter resulted in being more sensitive to this kind of treatment. It is interesting to note that pharmacological actions of CSDE resulted in being selective for cancer cell lines, as the treatment with increasing concentrations of CSDE (except for 100 μg/mL) did not affect the viability of normal prostate PNT2 cells. Moreover, the effect underlying the higher cytotoxicity effect observed in DU 145 cells was related to apoptosis phenomena because, according to Flow Cytometry analysis, after 24 h of incubation with 55.5 or 100 μg/mL of CSDE, the amount of cells in late apoptotic phase was higher if compared with control cells (cells treated with vehicle). Our findings are in agreement with Sorice et al.’s study [22] in which authors assessed the effect of polyphenols extracted from chestnut shells (PECS) on different cancer cell lines. The authors reported that, among the cancer cell lines used, treatment with increasing amounts of PECS only affected the viability of Hep G2 cells after 48 h of treatment. Moreover, authors also reported that PECS induced mitochondrial membrane depolarization, modification of cell cycle phases, modulation of tumor microenvironment by cytokines profiling, and modification of metabolite expression. Even if, in our experimental conditions, CSDE did not affect the viability of Hep G2 after 24 h of exposure, we also confirmed a cytotoxic effect on these cells after 48 h of CSDE treatment (data not shown) in accordance to the previous findings [22,35]. Particularly, our findings related to apoptosis analysis on DU 145 cells after CSDE treatment also confirm the results showed by Lee et al., in which chestnut extract displayed anti-proliferative and apoptosis induction in AGS gastric cells [9].

In conclusion, polyphenols compounds from CSDE showed significant cytotoxic and pro-apoptotic effects on several cancer cell lines of different origin and, in a particular manner, on DU 145 human prostate cancer cells—an in vitro model never tested with polyphenols from chestnut shells. Furthermore, our data demonstrate that a cheap and underused residue could be valorized through the production of a bioactive extract obtained by an eco-friendly experimental procedure. Even if our work represents a preliminary study on the role played by different polyphenols contained in the whole extract, our main objective will be to verify the further mechanistic actions triggering the apoptotic and cell survival pathways.

## 4. Materials and Methods

### 4.1. Chemicals

Chemicals for the phenolic classes determination (Folin–Ciocalteu reagent and Na_2_CO_3_ for phenolic compounds, HCl, NaNO_2_, and Na_2_MoO_4_ for *ortho*-diphenols, AlCl_3_∙6H_2_O for flavonoids, and cinchonine hemisulfate for tannins), HPLC standards, foetal bovine serum (FBS), penicillin/streptomycin, l-glutamine, 0.25% trypsin-EDTA, dimethylsulfoxide (DMSO), phosphate-buffed saline, and 3-(4,5-dimethylthiazol-2-yl)-2,5-diphenyltetrazolium bromide (MTT) were purchased from Sigma-Aldrich Co. (Milano, Italy). Methanol, acetic acid, and acetonitrile for HPLC analyses were from Carlo Erba Reagents (Cornaredo, Milano, Italy). Rosewell Park Memorial Institute 1640 medium (RPMI 1640), Dulbecco’s Modified Eagle Medium (DMEM), and Dulbecco’s Modified Eagle Medium/Nurient Mixture F12 (DMEM-F12) growth medium were obtained from Gibco by Life Technologies (Grand Island, NY, USA). All other chemicals were reagent grade and were obtained from commercial sources.

### 4.2. Extraction of Phenolic Compounds from Chestnut Shells

Burned inner and outer chestnut shells (CS) were kindly provided by Ingino s.r.l. food factory (Montoro Inferiore, Avellino, Italy). CS were dried in oven at 55 °C until reaching constant weight and powdered using a food homogenizer (type 8557-54, Tefal, France). Three different extractions, using water as a solvent, were carried out for the recovery of the bioactive molecules: 1) CLE in boiling water under continuous and vigorous stirring; 2) UAE at 60 °C in ultrasonic bath frequency 28–34 kHz, power 80–180 W, 230 V) (Ultrasonic Falc, Treviglio, Bergamo, Italy); 3) MAE (frequency 2450MHz, power 1000 W, input 1080 W, output 700 W). All the extractions were conducted for 60 min using 5% (*w*/*v*) CS. After the extraction process, each suspension was cooled on ice, centrifuged at 3220× *g* for 1 h at 4 °C (Eppendorf 5810R), and the supernatant was recovered. The remaining solid residue was rinsed with the same volume of water lost during the extraction, the resulting suspension was centrifuged as above, and the supernatant was added to the previous one in order to restore the original volume. The clear solutions were freeze-dried in an Edwards Modulyo freeze-dryer (Edwards, Cinisello Balsamo, Milano, Italy), and the powder chestnut shell dry extract (CSDE) was stored at room temperature (RT) until use.

### 4.3. Preparation of CSDE for Analyses 

A stock solution of 3 mg CSDE/mL was prepared in phosphate-buffered saline (PBS, pH 7.4) in order to perform the characterization of the extracts in the same vehicle used for the study on cancer cell lines. For cytotoxicity study, the CSDE was prepared immediately before use as a stock solution of 10 mg/mL in PBS.

### 4.4. Total Phenolic Content

The total phenolic content was measured by the Folin–Ciocalteu method [36]. Aliquots of samples, diluted to 150 μL with PBS, were mixed with 750 μL of Folin–Ciocalteu reagent (diluted ten-folds with deionized water) and 600 μL of 7.5% (*w*/*v*) Na_2_CO_3_. The reaction was developed at 25 °C for 2 h in the dark, and the absorbance was read at 765 nm against a blank prepared with 150 μL of PBS (Cary 100, Varian Analytical Instruments). The total phenolic content was estimated by a calibration curve prepared with increasing quantities of a standard solution of gallic acid (range 1.5–10 μg), and the results were expressed as mg GAE/g CSDE.

### 4.5. Total Ortho-Diphenolic Content

The *ortho*-diphenols were measured as described by Arnow [37]. Briefly, 100 μL of sample were diluted to 400 μL with PBS. Then, 400 μL of 0.5 M HCl, 400 μL of 1.45 M NaNO_2_, 0.4 M Na_2_MoO_4_, and 400 μL of 1 M NaOH were added in sequence. The absorbance of the resulting mixture was instantaneously measured at 500 nm against a blank prepared with 400 μL of PBS. The quantification was carried out by a calibration curve obtained with increasing quantities of a standard solution of caffeic acid (range 5–50 μg), and the results were expressed as mg CAE/g CSDE.

### 4.6. Total Flavonoid Content

The total flavonoid content was determined following the method of Barreira et al. [38] with some modifications. Briefly, 250 μL of phenolic extract were mixed with 1.25 mL of deionized water and 75 μL of 5% (*w*/*v*) NaNO_2_. After 5 min, 150 μL of 16% (*w*/*v*) AlCl_3_6H_2_O were added. After 1 min, 500 μL of 1 M NaOH and 275 μL of deionized water were added, and the resulting solution was vigorously mixed. The absorbance was read at 510 nm versus a blank containing 250 μL of PBS. The flavonoid amount was determined by a calibration curve obtained with increasing quantities of a standard solution of catechin (range 5–75 μg). The results were expressed as mg of catechin equivalents (CE)/g CSDE.

### 4.7. Total Tannin Content

The total tannin content was estimated according to Peri and Pompei [39], with some modifications. Briefly, 0.8 mL of the sample was added to 0.8 mL of 0.5% (*w*/*v*) cinchonine hemisulfate in a 2 mL Eppendorf tube. The solution was mixed for 5 min and left overnight at 4 °C in order to achieve a better precipitation. After centrifugation at 16,100× *g* for 5 min at 4 °C (Eppendorf 5415R), the supernatant, containing the non-tannin fraction, was separated from the pellet (tannin fraction). The Folin–Ciocalteu assay was carried out on the supernatant in order to calculate the non-tannin content. The total tannin fraction was determined by difference between the total phenolic content and the non-tannin content, and the results were expressed as mg GAE/g CSDE.

### 4.8. Reversed-Phase (RP)-HPLC Analysis 

RP-HPLC analysis was performed, as reported in Squillaci et al. [17]. The samples were dissolved in PBS at concentration of 10 mg/mL, filtered through a CHROMAFIL syringe filter (pore size 0.45 μm, Macherey-Nagel GmbH and Co., Duren, Germany), and injected into the column (50 μL). The peak elution was monitored at 280 nm. Phenolic compounds were identified by comparing the retention times with those of pure commercial standards, and by co-injection with the corresponding standards. Quantification was performed by calibration curves obtained by injecting increasing quantities of the pure compound, and the results were expressed as mg/g CSDE.

### 4.9. Cell Cultures

Human prostate cancer cell lines (PC-3, DU 145, and LNCaP), PNT2 (immortalized non-cancerous prostate epithelial cell line), MCF-7, and MDA-MB-231 (human breast cancer cells), and Hep G2 (hepatocellular cancer cell) were obtained from the American type culture collection (ATCC, Manassas, VA, USA). LNCaP and PNT2 cells were maintained in RPMI 1640 medium, while PC-3 cells were cultured in DMEM/F12. The other four cell lines were grown in DMEM. All the cells were supplemented with 10% FBS, 1% l-glutamine, 50 U/mL penicillin, 50 mg/mL streptomycin in the atmosphere of 5% CO_2_ in humified of 95% air at 37 °C.

### 4.10. Assessment of Cell Viability Assay

To determine whether CSDE exerted inhibitory activity towards the cell lines, the MTT assay was used. In brief, the cell lines were seeded in culture media which contained 10% FBS on a flat-bottomed 96-well plates at a density of 4000–15,000 cells per well. After 24 h of incubation, the cells were treated with increasing concentrations (1-3-10-17.5-30-55.5-100 μg/mL) of CSDE in a medium containing 1% FBS for 24 h. Control cells received only the vehicle (PBS). After the established incubation time, 100 μL of MTT (0.25 mg/mL) were added in each well according to the manufacturer’s instructions for 2–4 h. Thereafter, 100 μL of DMSO were added in each well. Absorbance (OD) at 570 nm was then measured using a microplate reader (BioTek™ Cytation™ 3, Winooski, VT, USA). The absorbance of the control wells was taken as 100%, and the results were expressed as a percentage of the control. The solutions were prepared on the same day of the experiments. 

### 4.11. Evaluation of Apoptosis by Flow Cytometry 

The apoptosis rate of DU 145 cells induced by CSDE was measured by using FCM. Cells were double stained with Kit Annexin V and PI apoptosis assay (Dojindo Molecular Technologies Inc., Munich, Germany). Cells were seeded into 100 mm dishes at density of 1 × 10^6^ in DMEM medium containing 10% FBS. When the cells reached 70% confluency, they were treated with 55.5 or 100 µg/mL of CSDE in a medium supplemented with 1% FBS for 24 h. After that, the cells were detached with trypsin and centrifuged at 400× *g* for 5 min, washed twice with 1 mL PBS, and the supernatant was discarded. Subsequently, the cells were resuspended with 400 μL of binding buffer. Then, 5 μL of Annexin V-PI was added into the suspension and incubated at RT for 15 min. Thereafter, 10 μL of PI staining solution was added, and further incubated for 5 min. The apoptosis rate of the cells was analyzed within 30 min with BD FACSCanto II system and elaborated using the DIVA software (BD Biosciences, Milan, Italy). For each condition, at least 10,000 events were recorded.

### 4.12. Statistical Analysis

All tests for the chemical characterization and statistical analysis for the cytotoxicity and apoptosis studies were performed by using GraphPad Prism 5.01 software (La Jolla, San Diego, CA, USA). Results are expressed as mean ± standard deviation (SD) of three independent experiments. One-way ANOVA followed by post hoc Tukey test was used to compare differences between experimental groups. Results with *p* < 0.05 were considered statistically significant.

## Figures and Tables

**Table 1:** Extraction yield, total phenols, and phenolic families in dry extracts from chestnut shells.

Extraction Method	Extraction Yield (*w*/*w*%) ^a^	Total Phenols (mg GAE/g CSDE) ^a,b,c^	*ortho*-Diphenols (mg CAE/g CSDE) ^a,d,c^	Flavonoids (mg CE/g CSDE) ^a,e,c^	Tannins (mg GAE/gCS DE) ^a,b,c^
CLE	5.2 ± 0.1	312.44 ± 3.32	148.72 ± 2.61	62.18 ± 1.19	205.99 ± 1.95
UAE	2.2 ± 0.1	190.12 ± 1.16	73.90 ± 1.01	47.75 ± 2.32	118.97 ± 2.12
MAE	3.8 ± 0.1	247.63 ± 3.42	104.20 ± 2.67	58.19 ± 1.18	175.91 ± 3.75

CLE = conventional liquid extraction; UAE = ultrasound-assisted extraction; MAE = microwave-assisted extraction. ^a^ All determinations were conducted in triplicate and results were expressed as mean ± SD values; ^b^ GAE: gallic acid equivalents; ^c^ CSDE: chestnut shell dry extract; ^d^ CAE: caffeic acid equivalents; ^e^ CE: catechin equivalents.

**Table 2:** Phenolic compounds identified by RP-HPLC in dry extracts from chestnut shells.

Compound	CLE (mg/g CSDE) ^a,b^	UAE (mg/g CSDE) ^a,b^	MAE (mg/g CSDE) ^a,b^
Gallic acid	86.97 ± 1.32	150.09 ± 2.16	117.58 ± 1.93
Protocatechuic acid	11.20 ± 0.30	21.57 ± 1.57	16.8 ± 0.09
Chlorogenic acid	0.67 ± 0.01	0.79 ± 0.05	1.18 ± 0.04
Epicatechin	0.71 ± 0.05	0.79 ± 0.04	1.28 ± 0.02
Syringic acid	0.2 0± 0.01	0.14 ± 0.01	0.21 ± 0.01
Ellagic acid	0.81 ± 0.06	0.58 ± 0.01	1.09 ± 0.03
*p*-Coumaric acid	0.22 ± 0.01	0.52 ± 0.02	0.43 ± 0.01
Sinapic acid	0.16 ± 0.02	0.48 ± 0.03	0.27 ± 0.01
Ferulic acid	0.03 ± 0.01	0.31 ± 0.05	0.09 ± 0.01
Scopoletin	0.11 ± 0.01	0.41 ± 0.03	0.20 ± 0.02

CLE = conventional liquid extraction; UAE = ultrasound-assisted extraction; MAE = microwave-assisted extraction. ^a^ All determinations were conducted in triplicate and results were expressed as mean ± SD values; ^b^ CSDE: chestnut shell dry extract.
